# Antimicrobial Activity and Modulatory Effect of Essential Oil from the Leaf of *Rhaphiodon echinus* (Nees & Mart) Schauer on Some Antimicrobial Drugs

**DOI:** 10.3390/molecules21060743

**Published:** 2016-06-08

**Authors:** Antonia Eliene Duarte, Irwin Rose Alencar de Menezes, Maria Flaviana Bezerra Morais Braga, Nadghia Figueiredo Leite, Luiz Marivando Barros, Emily Pansera Waczuk, Maria Arlene Pessoa da Silva, Aline Boligon, João Batista Teixeira Rocha, Diogo Onofre Souza, Jean Paul Kamdem, Henrique Douglas Melo Coutinho, Marilise Escobar Burger

**Affiliations:** 1 Centro de Ciências Biológicas e da Saúde (CC BS), Departamento de Ciências Biológicas, Universidade Regional do Cariri (URCA), Crato 63105-000, CE, Brazil; duarte105@yahoo.com.br (A.E.D.); flavianamoraisb@yahoo.com.br (M.F.B.M.B.); nadghia.fl@gmail.com (N.F.L.); lmarivando@hotmail.com (L.M.B.); arlene.pessoa@urca.br (M.A.P.d.S.); 2 Programa de Pós-Graduação em Bioquímica Toxicológica, Departamento de Bioquímica e Biologia Molecular, Universidade Federal de Santa Maria, Santa Maria 97105-900, RS, Brazil; memypw@yahoo.com.br (E.P.W.); alineboligon@hotmail.com (A.B.); jbtrocha@yahoo.com.br (J.B.T.R.); kamdemjeanpaul2005@yahoo.fr (J.P.K.); mariliseeb@yahoo.com.br (M.E.B.); 3 Molecular Chemistry and Pharmacology Laboratory, Universidade Regional do Cariri (URCA), Crato 63105-000, CE, Brazil; 4 Laboratório de Botânica Aplicada, Universidade Regional do Cariri (URCA), Crato 63105-000, CE, Brazil; 5 Departamento de Bioquímica, Instituto de Ciências Básica da Saúde, Universidade Federal do Rio Grande do Sul, Porto Alegre 90035-191, RS, Brazil; diogo@ufrgs.br; 6 Laboratory of Microbiology and Molecular Biology, Universidade Regional do Cariri (URCA), Crato 63105-000, CE, Brazil; hdmcoutinho@gmail.com; 7 Programa de Pós-Graduação em Farmacologia, Universidade Federal de Santa Maria, Santa Maria 97105-900, RS, Brazil

**Keywords:** natural products, *R. echinus*, antibacterial activity, antifungal activity, modulating

## Abstract

*Background*: *Rhaphiodon echinus* is a weed plant used in the Brazilian folk medicinal for the treatment of infectious diseases. In this study, the essential oil of *R. echinus* leaf was investigated for its antimicrobial properties. *Methods*: The chemical constituents of the essential oil were characterized by GC-MS. The antimicrobial properties were determined by studying by the microdilution method the effect of the oil alone, and in combination with antifungal or antibiotic drugs against the fungi *Candida albicans*, *Candida krusei* and *Candida tropicalis* and the microbes *Escherichia coli*, *Staphylococcus aureus* and *Pseudomonas*. In addition, the iron (II) chelation potential of the oil was determined. *Results*: The results showed the presence of β-caryophyllene and bicyclogermacrene in major compounds, and revealed a low antifungal and antibacterial activity of the essential oil, but a strong modulatory effect on antimicrobial drugs when associated with the oil. The essential oil showed iron (II) chelation activity. *Conclusions*: The GC-MS characterization revealed the presence of monoterpenes and sesquiterpenes in the essential oil and metal chelation potential, which may be responsible in part for the modulatory effect of the oil. These findings suggest that essential oil of *R. echinus* is a natural product capable of enhancing the antibacterial and antifungal activity of antimicrobial drugs.

## 1. Introduction

There is growing interest in providing a scientific basis for the therapeutic effects of natural products [1,2,3,4], since many people in the world depend on alternative medicines for their primary healthcare [5,6,7]. Antibiotic resistance is a natural phenomenon resulting from the modern selective pressure of the clinical use of antibiotics [8]. Reports have indicated that plants are good alternatives to synthetic chemical antimicrobials and antibiotics, which can cause: (i) serious side effects; (ii) antimicrobial resistance and (iii) the reemergence of previous infections due to the inadequate or widespread use of antimicrobials [9,10,11,12,13].

Invasive infections by yeast of the genus *Candida* are becoming increasingly important as a cause of infections in many hospitals due to medical progress itself, associated with the emergence of more invasive procedures, and the use of broad-spectrum antibiotics [14,15]. Nosocomial infections due to multidrug resistant Gram-negative bacteria have grown steadily, and are the major cause of morbidity and mortality worldwide [16]. Therapeutic options available for the treatment of acute bacterial infections require repeated administration and could result in prolonged hospitalization and substantial costs [17]. Hence, infections caused by multiple drug resistant bacteria are becoming important clinical problems in many countries [18]. Recently, reports have shown that treatments in combination with natural products may reduce the complications associated with multiple dosing, or enhance the effect of antibiotics [12].

Studies on the antimicrobial action of phytochemicals and their possible synergism with conventional antimicrobial drugs have thus generated considerable interest, since the synergism with plant extracts or phytochemicals against microbial strains was shown to be more effective than the conventional drug alone [19,20,21,22,23]. 

Iron is a micronutrient required by almost all living organisms, including fungi [24]. It is required for growth and proliferation, a feature of almost all organisms, except some bacteria [25,26]. The biological significance of iron lies in its ability to cycle between two oxidation states: the reduced (Fe^2+^, ferrous) and oxidized (Fe^3+^, ferric) forms. As a result, pathogenic microorganisms have developed a high affinity with iron. Of particular importance is the demonstration that some fungi have a metabolic demand for iron [27], and that iron overload can increase susceptibility to the infections caused by some fungi [28]. Interestingly, iron chelators can mobilize iron in tissues through the formation of soluble stable complexes, which are excreted in feces and/or urine. In this context, the use of iron chelators as therapeutic agents against different types of microbes can be of significant interest to reduce complications associated with iron overload, and thus improve the quality of life [29,30], making the search for natural products with potential antimicrobial activity and iron(II)-chelating ability of utmost importance [30].

The species *Rhaphiodon echinus* (Nees & Mart) Schauer is a plant of the family Lamiaceae, found in various environments, such as vacant lots and the caatinga area, that has been used in folklore medicine for treating cough, fatigue, pain, infections and inflammation [31]. Pharmacological studies on *R. echinus* indicated that it exhibits antimicrobial and antioxidant [32] activities, anti-inflammatory and analgesic [31] properties. Furthermore, a previous study revealed that essential oil from *R. echinus* leaf is rich in sesquiterpenes, mainly bicyclogermacrene and *trans*-caryophyllene [33]. More recently, Duarte *et al.* demonstrated that *R. echinus* aqueous and ethanolic extracts are rich in polyphenols and that the therapeutic effect of *R. echinus* may be, at least in part, attributed to its antioxidative activity.

Considering the challenge of antibiotic resistance and the promising combination therapy (antibiotic + phytochemical/plant extract) to overcome the present drug-resistant infectious microbial problem, this study sought to investigate *in vitro* the antimicrobial activity of *R. echinus* leaf essential oil and its possible interaction(s) with antimicrobial drugs against different strains of bacteria and fungi. In addition, the iron (II) chelation potential of the essential oil was investigated as well as its chemical constituents using GC-MS.

## 2. Results

### 2.1. Chemical Characterization of Essential Oil of R. echinus 

The yield of essential oil from the dried leaves of *R. echinus* was 0.12%. A total of 21 compounds were identified in the essential oil among which bicyclogermacrene (28.13%), β-caryophyllene (23.07%), caryophyllene oxide (5.40%), spathulenol (5.12%), α-camphene (4.09%), α-terpineol (3.76%), caryophyllene acetate (3.61%), and thymol (3.21%) are the major phytochemicals (Table 1). The oil showed a terpenic nature, containing mainly mono- and sesquiterpenes. Therefore, it is possible to assume that the strong odor of *R. echinus* leaf essential oil is due to the presence of monoterpenes as major components.

### 2.2. Assessment of Minimum Inhibitory Concentration of Essential Oil of R. echinus

The essential oil from the dried leaf of *R. echinus* showed a minimum inhibitory concentration (MIC) against strains of bacteria and fungi, higher or equal to 1024 μg/mL (data not shown). The MIC value > 1024 μg/mL is not relevant from the clinical point of view, hence, the essential oil was tested at sub-inhibitory concentrations in combination with standard antifungal and antibacterial drugs. 

### 2.3. Modulatory Effect of Essential Oil of R. echinus on Some Antifungal Drugs

**Figure 1:**
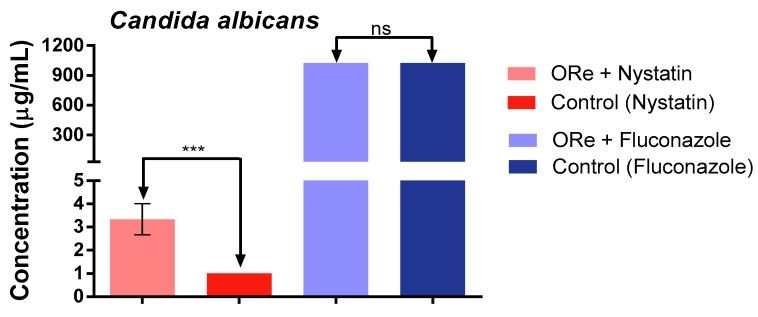
Minimum inhibitory concentration of nystatin and fluconazole in the presence and absence of *R. echinus* leaf essential oil for *Candida albicans*. ORe = *R. echinus* leaf essential oil. Statistical analysis: one-way ANOVA followed by Bonferroni *post-hoc* test. *** *p* < 0.001 indicates significant difference when *R. echinus* leaf essential oil was added to the medium. ns, not significant. Data show mean + SEM from three independent experiments performed in triplicate.

**Figure 2:**
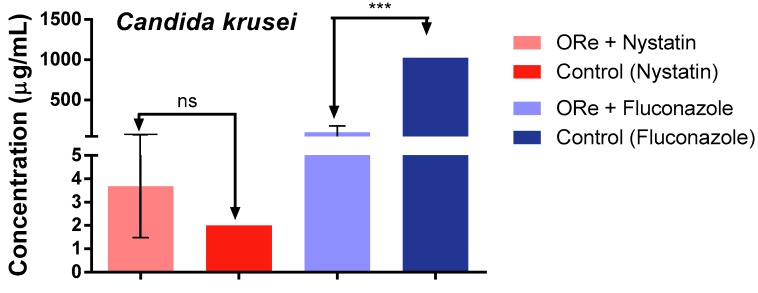
Minimum inhibitory concentration of nystatin and fluconazole in the presence and absence of *R. echinus* leaf essential oil for *Candida krusei*. ORe = *R. echinus* leaf essential oil. Statistical analysis: one-way ANOVA followed by Bonferroni *post-hoc* test. *** *p* < 0.001 indicates significant difference when *R. echinus* leaf essential oil was added to the medium. ns, not significant. Data show mean + SEM from three independent experiments performed in triplicate.

**Figure 3:**
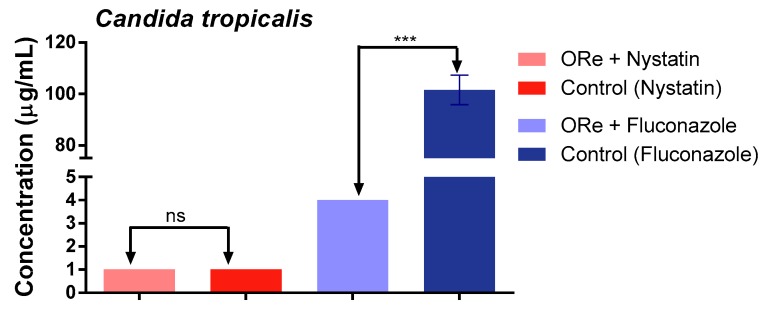
Minimum inhibitory concentration of nystatin and fluconazole in the presence and absence of *R. echinus* leaf essential oil for *Candida tropicalis*. ORe = *R. echinus* leaf essential oil. Statistical analysis: one-way ANOVA followed by Bonferroni *post-hoc* test. *** *p* < 0.001 indicates significant difference when *R. echinus* leaf essential oil was added to the medium. ns, not significant. Data show mean + SEM from three independent experiments performed in triplicate.

Figure 1, Figure 2 and Figure 3, depict the effect of combination treatment of essential oil of *R. echinus* leaf with some antifungal drugs (nystatin and fluconazole) on *Candida albicans* (Figure 1), *C. krusei* (Figure 2) and *C. tropicalis* (Figure 3). There was a significant antagonistic association between the essential oil and nystatin in comparison with nystatin alone (Figure 1, *p* < 0.001). However, no synergistic effect was observed between the essential oil and nystatin against *C. krusei* (Figure 2) and *C. tropicalis* (Figure 3) in comparison with nystatin alone (*p* > 0.05). Interestingly, the addition of the essential oil to the growth medium (at the sub-inhibitory concentration) in the presence of fluconazole showed synergistic action against *C. krusei* (Figure 2) and *C. tropicalis* (Figure 3) in comparison with fluconazole alone (*p* < 0.001). This was evidenced by growth inhibition at lower concentration in comparison with that of the antifungal drug fluconazole (Figure 2 and Figure 3). In contrast, the combination of fluconazole and the essential oil did not have any effect on *C. albicans* (*p* > 0.05; Figure 1).

### 2.4. Modulatory Effect of Essential Oil of R. echinus on Some Antibacterial Drugs

**Figure 4:**
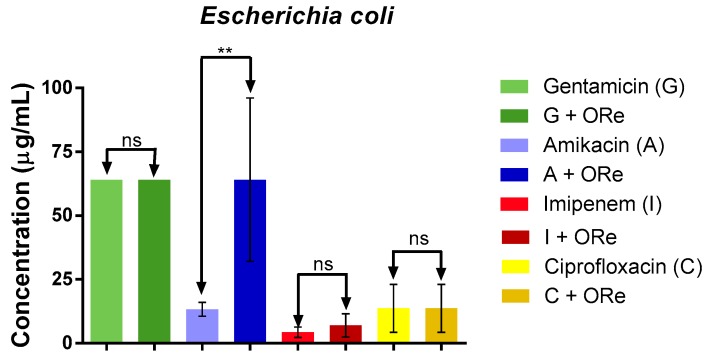
Minimum inhibitory concentration of antibiotics in the presence and absence of *R. echinus* leaf essential oil for *Escherichia coli*. ORe = *R. echinus* leaf essential oil. Statistical analysis: one-way ANOVA followed by Bonferroni *post-hoc* test. ** *p* < 0.01 indicates significant difference when *R. echinus* leaf essential oil was added to the medium. ns, not significant; G = gentamicin, A = amikacin, I = imipenem, C = ciprofloxacin. Data show mean + SEM from three independent experiments performed in triplicate.

**Figure 5:**
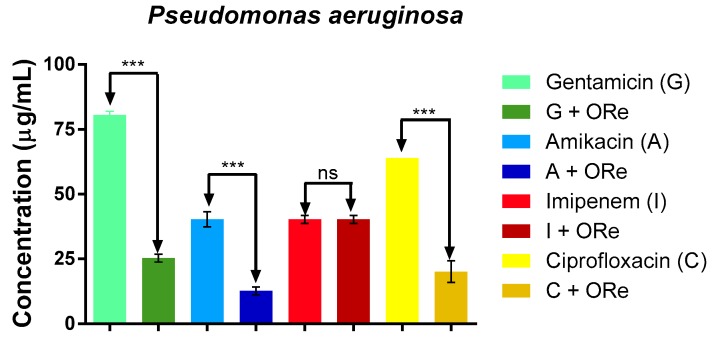
Minimum inhibitory concentration of antibiotics in the presence and absence of *R. echinus* leaf essential oil for *Pseudomonas aeruginosa*. ORe = *R. echinus* leaf essential oil. Statistical analysis: one-way ANOVA followed by Bonferroni *post-hoc* test. *** *p* < 0.001 indicates significant difference when *R. echinus* leaf essential oil was added to the medium. ns, not significant; G = gentamicin, A = amikacin, I = imipenem, C = ciprofloxacin. Data show mean + SEM from three independent experiments performed in triplicate.

**Figure 6:**
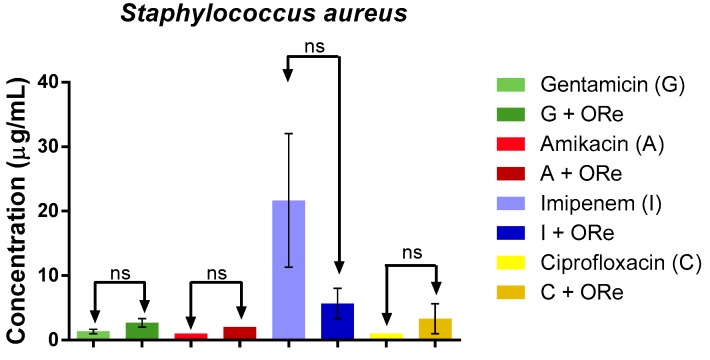
Minimum inhibitory concentration of antibiotics in the presence and absence of *R. echinus* leaf essential oil for *Staphylococcus aureus*. Statistical analysis: one-way ANOVA followed by Bonferroni *post-hoc* test, ns, not significant. ORe = *R. echinus* leaf essential oil. Ns, indicates significant difference when *R. echinus* leaf essential oil was added to the medium. G = gentamicin, A = amikacin, I = imipenem, C = ciprofloxacin. Data show mean + SEM from three independent experiments performed in triplicate.

The potential modulatory effect of the essential oil of *R. echinus* on some standard antibiotics (gentamicin (G), amikacin (A), imipenem (I) and ciprofloxacin (C)) against *Escherichia coli*, *Pseudomona aeruginosa* and *Staphylococcus aureus* was as shown in Figure 4, Figure 5 and Figure 6, respectively. The study revealed that combination of the essential oil with the antibacterial drugs caused a significant reduction in the MIC value only with amikacin (against *E. coli*), when compared with amikacin alone (Figure 4, *p* < 0.01); suggesting a synergistic effect with amikacin. However, the combination of the essential oil with gentamicin, amikacin, imipenem or ciprofloxacin did not exhibit any significant action against *S. aureus* (Figure 6, *p* > 0.05). As depicted in Figure 5, there was a significant synergistic action when the combination of the essential oil with gentamicin, amikacin and ciprofloxacin was used against *P. aeruginosa* in comparison with their respective controls (*p* < 0.001); however, synergism was not observed with imipenem against *P. aeruginosa* (Figure 5).

### 2.5. Fe^2+^ Chelation or Oxidation Potential of Essential Oil from R. echinus

**Figure 7:**
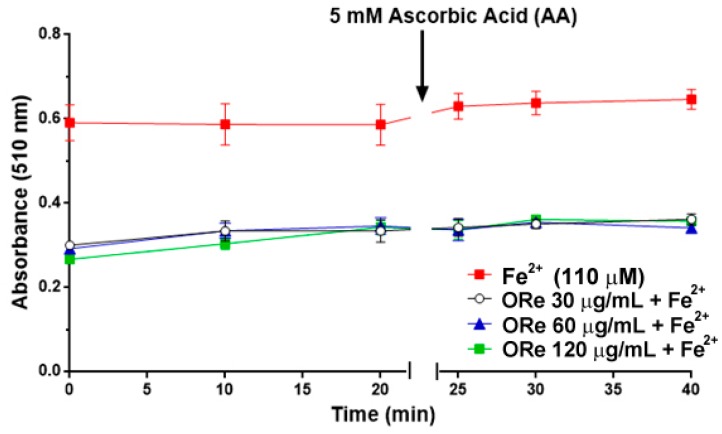
Oxidation of Fe^2+^ by *R. echinus* leaf essential oil (30–120 μg/mL). The essential oil was incubated with FeSO_4_ (110 μM) for 10 min. Then, *ortho*-phenanthroline was added and the absorbance of the reaction was measured at 0, 10 and 20 min following its addition of *ortho*-phenanthroline. After the last reading (at 20 min), 5 mM of ascorbic acid (AA) was added and the absorbance was read again at 5 min, 10 min and 20 min. Values represent the mean ± SEM of 3 independent experiments performed in duplicate. ORe = *R. echinus* leaf essential oil.

In this iron chelation assay, the rate of reduction in the absorbance of an orange coloured complex formed by Fe^2+^ and *ortho*-phenanthroline allows the estimation of a co-existent chelator. As seen in Figure 7, essential oil from the leaves of *R. echinus* (30–120 μg/mL) caused a decrease in the absorbance, suggesting possible Fe^2+^ chelating properties. This effect was observed for all the concentrations of the essential oil tested.

However, in order to investigate whether the reduction in absorbance in the presence of the essential oil was attributable to Fe^2+^ chelation or oxidation, ascorbic acid (AA) was added to the reaction medium 20 min after incubating Fe^2+^ with the essential oil and *ortho*-phenanthroline to reduce any Fe^3+^ that could have been formed. Addition of AA into the reaction medium did not change the absorbance after 5, 10 and 20 min, suggesting that the essential oil stimulated Fe^2+^ chelation during the incubation times (before AA addition) (Figure 7).

## 3. Discussion

In the current study, the essential oil from *R. echinus* leaves was tested alone or in combination with standard antifungal and antibacterial drugs against different strains of fungi and bacteria. The results showed the essential oil possesses the ability to modulate the actions of some antimicrobial drugs against some bacteria and fungi strains. The synergistic action observed can be partly attributed to the triterpenoid constituents of the essential oil [33,35]. There are few studies in the literature that show the chemical profile of essential oil *R. echinus*. In study of Torres *et al.* the chemical composition of essential oil from both the leaves and fruits of *R. echinus* was evaluated by GC-MS and GC-FID and 19 compounds (93.8% in the leaf oil and 82.4% in the fruit oil) were identified, mostly sesquiterpenes, including bicyclogermacrene and *trans*-caryophyllene. However, there exist some differences in the phytoconstituents characterized in this present study when compared to those reported by Torres *et al.* [33]. Such differences can be attributed to intrinsic factors such as genetics and specificity or extrinsic factor, such as environmental conditions, and the time and place of harvest or collection.

With the increasing incidence of antibiotic resistance, exploration of natural products from plants represents an interesting alternative therapy [36], since they may modulate the action of antibiotics, either by increasing or decreasing their activities [37]. Essential oils of many plants have shown that in addition to their antibacterial properties, they also possess the ability to interfere with antibiotic activity and exhibit a strong trend in the potentiation of antibiotics [36,38]. However, the results from this finding indicated that the studied essential oil showed no antifungal and antibacterial activity against multidrug-resistant microbial strains (MIC value ≥ 1024 μg/mL). Furthermore, the activity of some antifungal agents was potentiated (synergistic effect) upon the addition of the essential oil into the growth medium at sub-inhibitory concentrations (*i.e.*, MIC/8 = 128 μg/mL). A previous study has revealed that essential oils from some species of the Lamiaceae family including *Menta arvensis*, *Menta piperita*, *Mentas picata* and *Origanum vulgare* have strong anti-Candida activity [39]. Bicyclogermacrene, sesquiterpenoids and other terpenoids, which were the major classes of phytochemicals found in this essential oil have also been identified in the essential oil of *Piper cernuum* and *Piper reginelli* where they demonstrated antimicrobial activity [40,41].

The antimicrobial activity of gentamicin, ciprofloxacin and amikacin against resistant strains of *P. aeruginosa* was significantly modulated by the essential oil when added at a sub-inhibitory concentration (MIC/8 = 128 μg/mL). However, the essential oil antagonized the antibacterial effect of amikacin and imipenem against *E. coli* and did not have any effect against *S. aureus*. A natural explanation for this phenomenon may be due to differences in the bacterial cell wall structures of both bacterial groups. Diterpenes found in the essential oil have been reported as modifiers of antibiotic activity [42], suggesting that its use could represent an advance against drug resistance mechanisms [43]. Hence, combination of terpenes and antibiotics offers a promising alternative strategy for the treatment of infectious diseases. 

Generally, the antimicrobial mechanism of action of natural compounds includes disintegration of cytoplasmic membranes, destabilization of the proton motive force (MPF), electron flow, active transport and coagulation of the cell contents. An important feature responsible for the antimicrobial activity of essential oils could be the hydrophobicity of their components which favors the alteration of the bacterial cell membrane, making it more permeable [44] or causing osmotic changes [45]. It should be stressed that in another set of experiments, we observed that *R. echinus* leaf essential oil caused membrane disruption of *Trypanosoma cruzi* and *Leishmania* species (data under consideration elsewhere). Therefore, it seems that the membrane disruption caused by the essential oil might be one of the mechanism(s) by which the oil component(s) acted in the present study, but this possibility was not investigated here, and may thus constitute a limitation of this study. Components of essential oils could also act on the cytoplasmic membrane proteins [46]. Cyclic hydrocarbons may act on ATPases that are known to be located in the cytoplasmic membrane and surrounded by lipid molecules. The lipid hydrocarbons could distort lipid-protein interaction, and direct interaction of lipophilic compounds with hydrophobic parts of the protein [47]. Some essential oils stimulate the growth of pseudomycelium, an indication that they may act on the enzymes involved in the synthesis of structural components of bacteria [48]. 

Iron is an element critical for normal growth and development [49,50], and the most abundant transition metal in the body [51]. However, free iron accumulation (II) as a result of oxidative stress has been found in many neurological disorders, including Alzheimer’s disease and Parkinson’s disease [1,52]. Therefore, chelation therapy to reduce complications associated with iron became a therapeutic strategy for the treatment of Alzheimer’s disease [1,53]. In this study, we evaluated the chelating property of the essential oil by monitoring its interfere with Fe^2+^-*ortho*-phenanthroline complex formation, as evidenced by the decrease in absorbance before the addition of ascorbic acid. Hence, the Fe^2+^ chelating ability of the essential oil was confirmed by the maintenance of the absorbance after the addition of ascorbic acid (Figure 7). This is consistent with an earlier study which demonstrated Fe^2+^ chelating ability of essential oil from lemon peels with this property linked to its constituent phytochemicals [30]. Thus, the modulatory effect of the essential oil of *R. echinus* on the selected antimicrobial drugs could be associated with its Fe^2+^ chelating ability of its constituent phytochemicals. A study reported that plant-derived phytochemicals with the capacity to prevent lipid oxidation and microbial spoilage have tremendous potential to extend the shelf life of food products with minimal use of synthetic preservatives [54]. The chelating ability can be associated with low growth through chelation of nutrient Mn^2+^ and Zn^2+^ an activity that results in antimicrobial effects. Thus, screening using a chelating assay for Fe^2+^, can indicate one possible strategy for microbial inhibition, as shown in the Corbin *et al.* [55]. Studies that corroborate our results have also demonstrated Fe^2+^ chelating ability of other essential oils as *Origanum vulgare* L., *Thymus vulgaris* L., *Rosmarinus officinalis* L., *Syzygium aromaticum* (L.) Merril & Perry, *Mentha spicatha* L., *Mentha crispa* L. and *Mentha piperita* L. [56,57].

## 4. Materials and Methods 

### 4.1. Plant Material 

The leaves of *R. echinus* were collected in Crato, Ceará, Brazil. The plant material was identified by Maria da Silva and a specimen was deposited at the Herbarium Caririense Dárdano de Andrade-Lima, Regional University of Cariri (URCA).

### 4.2. Preparation of Essential Oil from Raphiodon echinus (Nees e Mart) Shauer Leaf

The essential oil of *R. echinus* was extracted from dried plant material subjected to hydro-distillation in a Clevenger apparatus. Briefly, the leaves were dried under the sun, crushed into small pieces and put in a 1 L volumetric flask, and 300 mL of distilled water was added. The flask was attached to a Clevenger apparatus placed on a heating mantle and the dried leaves were boiled. After each extraction cycle, the oil contained in the apparatus was collected with a pipette, stored in amber bottles, and refrigerated. At the end of the extraction process, the oil was dried over anhydrous sodium sulfate to remove the aqueous phase, and stored at 4 °C prior to use. 

### 4.3. Analysis of Chemical Composition of the Essential Oil of Rhaphiodon echinus by Gas Chromatography Coupled with Mass Spectrometry (GC-MC) 

The gas chromatography (GC) analysis was performed with an Agilent Technologies 6890N GC-FID system (Santa Maria-RS, Brazil), equipped with DB-5 capillary column (30 m × 0.32 mm; 0.50 mm) and connected to an FID detector. The thermal programmer was 60 °C (1 min) to 180 °C at 3 °C/min; injector temperature 220 °C; detector temperature 220 °C; split ratio 1:10; carrier gas helium; flow rate: 1.0 mL/min. The injected volume was 1 μL diluted in chloroform (1:10). Two replicates of samples were processed in the same way. Component relative concentrations were calculated based on GC peak areas without using correction factors [58].

GC-MS analyses were performed on an Agilent Technologies AutoSystem XL GC-MS system operating in the EI mode at 70 eV, equipped with a split/splitless injector (220 °C). The transfer line temperature was 220 °C. Helium was used as carrier gas (1.0 mL/min) and the capillary columns used were an HP 5MS (30 m × 0.35 mm; film thickness 0.50 mm) and an HP Innowax (30 m × 0.32 mm i.d., film thickness 0.50 mm). The temperature programmer was the same as that used for the GC analyses. 

Identification of the constituents was performed on the basis of retention index (RI), determined with reference of a homologous series of C_7_-C_30_ *n*-alkanes, under identical experimental conditions, comparisons with the results mass spectra library searches (Adams, NIST and Wiley), and with the mass spectra literature data [34]. The relative amounts of individual components were calculated based on the GC peak area (FID response).

### 4.4. Drugs

The tested antibiotics were amikacin, gentamicin, ciprofloxacin and imipenem (Sigma Co., St. Louis, MO, USA). Antifungal drugs used were nystatin (Laboratorio Teuto Brasileiro, S/A, Anápolis, Brazil) and fluconazole (Prati, Donaduzzi& Cia Ltd., Toledo, Brazil). All the solutions were prepared following the recommendations of the National Committee for Clinical Laboratory Standards—NCCLS [59].

### 4.5. Strains of Bacteria and Growth Media

The bacterial strains used in this study were: *Escherichia coli* (EC-EC27), *Staphylococcus aureus* (SA358) and *Pseudomonas aeruginosa* (PA 03). They were obtained from the Clinical Microbiology Laboratory of the Federal University of Paraíba (João Pessoa, PB, Brazil), and maintained on nutrient agar slants at 4 °C. Prior to assay, the cells were cultured for 24 h in Heart infusion agar (Difco Laboratory, Ltd., Detroit, MI, USA) and medium brain heart infusion (BHI, Difco Laboratories Inc.). The source of the bacterial strains and their resistance to antibiotics are listed in Table 2. 

### 4.6. Strains of Fungi

The fungal strains used were: *Candida albicans* (CA-LM 62), *Candida tropicalis* (CT LM 23) and *Candida krusei* (CK LMBM 02). All strains were maintained on slants with Sabouraud Dextrose Agar (SDA, Difco Inc.), and before testing, the cells were subcultured for 24 h at 37 °C again in SDA. All strains were obtained from the Mycology Laboratory Clinic of the Federal University of Paraíba (João Pessoa, PB, Brazil) and held on SDA slants at 4 °C.

### 4.7. Matrix Solution Preparation

The essential oil of *R. echinus* leaf was firstly diluted in DMSO and then, sterile distilled water was added to avoid any effect of DMSO on the microorganisms. The concentration of the matrix solution was 2.048 μg/mL.

### 4.8. Minimum Inhibitory Concentration 

The minimum inhibitory concentration (MIC) was determined in 10% BHI (bacteria) and Sabouraud Dextrose Broth (Difco Ltd.) (*Candida* yeasts) by the microdilution method. The MIC is defined as the lowest concentration at which no microbial growth is observed. Briefly, 100 μL of culture medium containing different bacterial and fungal strains (10^5^ CFUs/mL, 10%) were distributed in 96-well plates and then diluted serially in 100 μL of varying concentrations (from 1024 to 2 μg/mL) of the essential oil [60]. The assay was performed concomitantly with the standards antifungal and antibacterial drugs. The antibacterial activity of the essential oil was detected by adding 20 μL of 0.01% aqueous solution of resazurin in each well at the end of the incubation period. Bacterial growth was monitored by irreversible reduction of resazurin, characterized by a change in color from blue to pink. The antifungal activity of the essential oil was determined by observing the turbidity, which is indicative of fungal growth. 

### 4.9. Modulatory Effect of Essential Oil from R. echinus on Antimicrobial Drugs

To evaluate the potential modulatory effect of the essential oil on microbial resistance; the oil was tested at the sub-inhibitory concentration (*i.e.*, MIC/8) in combination with antimicrobial drugs. One hundred microlitres (100 μL) of solution containing culture medium, the inoculum (10%) and the essential oil was distributed in alphabetical order in each well of the plate. Then 100 μL of antibacterial or antibiotic standard drugs was added and mixed. The final concentrations of antimicrobial standard drugs used in the present study ranged from 0.5—512 μg/mL. The plates were incubated for 24 h at 37 °C [37,61]. It should be stressed that for this set of assay, multiresistant bacteria was used.

### 4.10. Iron Chelating Property of Essential Oil from R. echinus 

The Fe^2+^ chelating property of the essential oil was determined using a modified method of Kamdem *et al.* [62]. A reaction mixture containing saline solution (58 μL, 0.9%, *w*/*v*), Tris-HCl (45 μL, 0.1 M, pH, 7.5), the oil (27 μL, 30–120 μg/mL) and 110 μM FeSO_4_ (36 μL) was incubated for 10 min at 37 °C. Subsequently, 1,10-phenanthroline (34 μL, 0.25%, *w*/*v*) was added and the absorbance of the orange colored complex formed was measured at 0, 10 and 20 min at 510 nm (against blank solutions of the samples) using microplate reader SpectraMax (Molecular Devices, Orleans Drive Sunnyvale CA, USA). The same procedure was performed for the control (*i.e.*, Fe^2+^), but without the extract. To ascertain the chelating potential of the oil, we determined the potential reduction of any Fe^3+^ (that might be formed during the incubation periods) by adding the reducing agent, ascorbic acid (to give a final concentration of 5 mM) to the reaction mixture. The absorbance was then determined after 5, 10 and 20 min following ascorbic acid addition. This is because the extracts could be oxidizing Fe^2+^ to Fe^3+^, leading to a decrease in absorbance that was not related to Fe^2+^ chelation. 

### 4.11. Statistical Analysis

Results are expressed as mean ± standard error of the mean (SEM). Statistical analysis was performed using two-way (ANOVA) followed by Bonferroni *post-hoc* test and *p* < 0.05 was considered statistically significant.

## 5. Conclusions 

In conclusion, this study demonstrated for the first time the modulatory effect of essential oil of *R. echinus* leaves on selected antimicrobial drugs used against different strains of fungi and bacteria, as well as its Fe^2+^ chelating ability. However, further studies on its isolated constituents need to be investigated alone and/or in combination with standard antimicrobial drugs, to be able to unravel possible synergistic effects, which could be employed against the treatment of multidrug resistance microbial infection in the future. Nevertheless, *R. echinus* appears to be a useful source of natural products, which may provide some protection against oxidative damage and food spoilage.

## Figures and Tables

**Table 1:** GC-MS analysis of the composition of essential oil of *R. echinus* leaf.

Compounds	RI ^a^	RI ^b^	Essential Oil (%)
α-Pinene	940	939	0.85
α-Camphene	951	953	4.09
β-Pinene	985	980	0.05
α-Phellandrene	1007	1005	2.11
α-Terpinene	1019	1019	0.28
*p*-Cymene	1032	1029	3.02
1,8-Cineole	1031	1033	1.19
γ-Terpinene	1060	1061	0.18
Methyl benzoate	1091	1091	0.92
α-Terpineol	1185	1189	3.76
Isoborneol	1156	1156	1.49
Geraniol	1258	1255	0.36
Thymol	1291	1290	3.21
γ-Elemene	1342	1339	2.87
Geranyl acetate	1387	1383	1.43
Dodecanal	1411	1407	0.65
β-Caryophyllene	1419	1418	23.07
Geranyl propionate	1476	1475	0.24
Germacrene D	1481	1480	3.16
Bicyclogermacrene	1495	1494	28.13
Germacrene A	1500	1503	1.76
β-Curcumene	1513	1512	0.08
Spathulenol	1577	1576	5.12
Caryophyllene oxide	1581	1579	5.40
Globulol	1584	1583	1.39
α-Cadinol	1653	1653	0.82
Caryophyllene acetate	1702	1700	3.61
Total identified (%)			99.24

Relative proportions of the essential oil constituents were expressed as percentages. ^a^ Retention indices from experimental analysis (based on homologous series of C_7_-C_30_ *n*-alkanes). ^b^ Retention indices [34].

**Table 2:** Bacterial resistance profile against antibiotics.

Bacteria	Source	Resistance Profile
*Escherichia coli* 27	Surgical Wound	Ast, Ami, Amox, Ca, Cfc, Cf, Caz, Cip, Clo, Im, Can, Szt, Tet, Tob
*Staphylococcus aureus* 358	Surgical Wound	Oxa, Gen, Tob, Ami, Can, Neo, Para, But, Sis, Net
*Pseudomonas aeruginosa* 03	Catheter tip	Cpm, Ctz, Im, Cip, Ptz, Lev, Mer, Ami

Ast—Aztreonan; Amp—Ampicillin; Ami—Amikacin; Amox—Amoxicillin; But—Butirosin; Ca—Cedroxil; Cfc—Cefaclor; Cf—Cephalothin, Caz—Ceftazinidime; Cpm—Cefepime; Ctz—Ceftazidime, Cip—Ciproflaxacin; Clo—Chloramphenicol; Can—Kanamycin; Gen—Gentamicin; Im—Imipenem; Lev—Levofloxacin; Mer—Meropenem; Neo—Neomycin; Net—Netilmicin; Oxa—Oxacillin; Par—Paromomycin; Ptz—Piperacillin-Tazobactam; Szt—Sulphametrim; Sis—Sisomicin; Tet—Tetraciclin; Tob—Tobromicina.
